# Cryo-EM structure of a thermostable bacterial nanocompartment

**DOI:** 10.1107/S2052252521001949

**Published:** 2021-04-02

**Authors:** Timothy Wiryaman, Navtej Toor

**Affiliations:** a Department of Chemistry and Biochemistry, University of California, San Diego, 9500 Gilman Drive, La Jolla, CA 92093, USA

**Keywords:** cryo-electron microscopy, nanocompartments, encapsulins, thermostability

## Abstract

Protein nanocompartments are widespread in bacteria and archaea, but their functions are not yet well understood. Here, the cryo-EM structure of a nanocompartment from the thermophilic bacterium *Thermotoga maritima* is reported at 2.0 Å resolution. The high resolution of this structure shows that interactions in the E-loop domain may be important for the thermostability of the nanocompartment assembly. Also, the channels at the fivefold axis, threefold axis and dimer interface are assessed for their ability to transport iron. Finally, an unexpected flavin ligand was identified on the exterior of the shell, indicating that this nanocompartment may also play a direct role in iron metabolism.

## Abstract 2

A 2.0 Å resolution cryo-EM structure of a thermostable bacterial nanocompartment is reported, with the high-resolution structure allowing the visualization of key details and new proposed biological functions.

## 1. Introduction   

Many species of bacteria and archaea have nanocompartments, which are protein assemblies that serve as non-membranous compartments (Nichols *et al.*, 2017 ▸; Giessen & Silver, 2017 ▸). These bacterial nanocompartments, which are also known as encapsulins, strongly resemble icosahedral viral capsids in structure, although there is little sequence conservation between these complexes (Sutter *et al.*, 2008 ▸). Aside from their structural similarities, the size and symmetry of encapsulins can vary to allow different assemblies and volumes (Giessen *et al.*, 2019 ▸). One major difference between encapsulins and viral capsids is that bacterial nanocompartments have binding sites in the interior for a specific peptide tag found on the C-terminus of cargo proteins (Sutter *et al.*, 2008 ▸; Cassidy-Amstutz *et al.*, 2016 ▸). These cargo proteins generally catalyze reactions that remove toxic metabolites or reactions involving toxic substrates, intermediates or products (Giessen & Silver, 2017 ▸). The cargo proteins are diverse and include dye-decolorizing peroxidases (DyPs), ferritin-like proteins, iron-mineralizing encapsulin-associated firmicute (IMEF) proteins, hemerythrins, copper nitrite reductase/hydroxyl­amine oxidoreductase fusion proteins, cysteine desulfurases and polyprenyl synthetases (Giessen & Silver, 2017 ▸; Nichols *et al.*, 2020 ▸; Tracey *et al.*, 2019 ▸).

**Figure 1:**
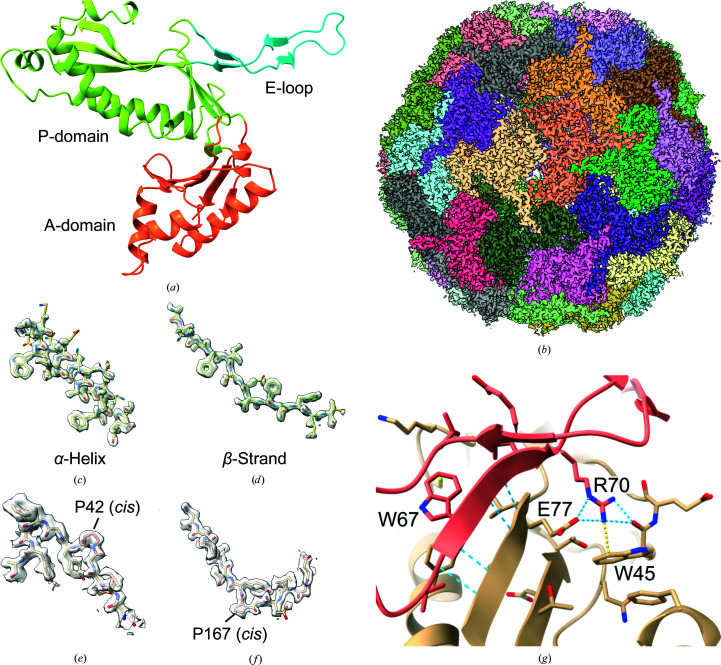
Density maps and model of TmEnc. (*a*) Domain organization of an encapsulin monomer, as viewed from the inside of the compartment. (*b*) Cryo-EM density map for the entire encapsulin nanocompartment. Subunits are colored individually. (*c*) Representative cryo-EM density for an α-helix (residues 14–30). (*d*) Representative density for a β-strand (residues 241–254). (*e*, *f*) Density for two *cis*-prolines, which were previously not observed. (*g*) Intermolecular interactions between the E-loop domain (tan) and another subunit (pink). Blue dashed lines indicate hydrogen bonding, and the yellow dashed line is a cation–π interaction.

Based on their unique assembly and storage properties, bacterial nanocompartments have been engineered for several different applications, such as catalytic nanoreactors, bio­logical reporters, targeted delivery systems, and nanomaterials and biomaterials (Jones & Giessen, 2021 ▸). A structural model of a nanocompartment is useful for designing nanocompartments for these kinds of applications. The first X-ray crystal structure of an encapsulin from *Thermotoga maritima* was determined in 2008 at 3.1 Å resolution (Sutter *et al.*, 2008 ▸). This structure demonstrated the structural similarity of encapsulins to the HK97 major capsid protein. The structure is divided into three domains: the major P-loop domain, which is a mixed α–β structure, the E-loop, which forms the twofold interactions, and the A-domain, which forms the fivefold symmetry axis interface [Fig. 1 ▸(*a*)]. Also, the structure contained additional density that was identified as the C-terminal extension of the cargo protein.

In this article, we report the 2.0 Å resolution cryo-electron microscopy (cryo-EM) structure of the thermostable nanocompartment from *T. maritima* [Fig. 1 ▸(*b*)]. At this resolution, we were able to model amino-acid side chains confidently and identify ordered solvent and ion molecules. The structure reveals interactions that are likely to be responsible for the thermostability of this complex relative to its mesophilic counterparts. Also, we examined possible iron-transport channels, particularly at the fivefold and threefold axes. Finally, we identified the binding site for an unexpected flavin ligand in the shell, which may suggest additional functions of encapsulins in iron metabolism.

## 2. Methods   

### 2.1. Protein expression and purification   

A codon-optimized gene coding for *T. maritima* encapsulin (TmEnc) was inserted into pET-11a and transformed into *Escherichia coli* Rosetta2 (DE3) cells (Novagen). 2 ml lysogeny broth with 100 µg ml^−1^ carbenicillin and 25 µg ml^−1^ chloramphenicol was inoculated with the strain expressing TmEnc and grown to an OD_600_ of 0.6 at 37°C in a rotary shaker. 1 l of autoinduction medium with carbenicillin and chloramphenicol was inoculated with 1 ml of the starter culture, incubated at 37°C to an OD_600_ of 2.9 with shaking and then transferred to 20°C for overnight protein expression. The cells were centrifuged at 5000*g* for 15 min at 4°C and resuspended in Enc buffer (50 m*M* Tris pH 8.0, 150 m*M* sodium chloride). The cells were lysed by sonication and the cell debris was centrifuged at 12 000*g* for 30 min at 4°C. Calcium chloride at a final concentration of 5 m*M* and 50 µl micrococcal nuclease (New England Biosciences) were added to the lysate and incubated at 37°C for 3 h to degrade nucleic acids. The lysate was incubated at 80°C for 1.5 h and centrifuged at 12 000*g* for 30 min at 4°C to remove the host proteins. Solid ammonium sulfate was added to 50% saturation slowly at 4°C and incubated for 1 h with stirring to remove the remaining host proteins. After centrifugation, the ammonium sulfate precipitation was repeated to 75% saturation to precipitate TmEnc [Supplementary Fig. S1(*a*)]. The pellet was resuspended in Enc buffer and purified by gel filtration on a HiPrep Sephacryl S-500 HR column (GE Healthcare Life Sciences) [Supplementary Fig. S1(*b*)]. UV–Vis spectra were collected with a Nanodrop spectrophotometer (Thermo Fisher) in triplicate [Supplementary Fig. S1(*c*)].

### 2.2. Grid preparation and cryo-electron microscopy data collection   

Copper 300-mesh Quantifoil 1.2/1.3 grids were plasma-cleaned with a Solarus plasma-cleaning system (Gatan). Grid preparation was performed with an EM GP automatic plunge freezer (Leica Microsystems). The chamber was set to 95% humidity at 20°C. 3 µl TmEnc at 2 mg ml^−1^ was applied to the grids, immediately blotted for 5 s and plunged into liquid propane. Cryo-electron microscopy data were collected on a Titan Krios (Thermo Fisher) at 81 000× magnification at a defocus of 0.5–2.5 µm. 2772 movies consisting of 67 frames were captured over 2.5 s on a K3 camera (Gatan) with an exposure rate of 0.5 e^−^ Å^−2^ per frame (Table 1 ▸).

### 2.3. Cryo-EM data processing   

All software was compiled and distributed by SBGrid (Morin *et al.*, 2013 ▸). Data processing was performed with *RELION* 3.1 (Scheres, 2012 ▸). Movies were motion-corrected with the internal *MotionCorr* implementation in *RELION* and CTF parameters were estimated with *CTFFIND*4 [Supplementary Fig. S1(*d*)] (Rohou & Grigorieff, 2015 ▸). Particles were picked with *crYOLO* using 1000 manually picked particles to train a model [Supplementary Fig. S1(*e*)] (Wagner *et al.*, 2019 ▸). After the first 3D refinement, beam-tilt refinement, particle polishing, defocus refinement and higher-order aberration correction were applied (Zivanov *et al.*, 2020 ▸) to further improve the reconstruction to a final resolution of 2.0 Å [Supplementary Fig. S1(*f*)] (Table 1 ▸).

### 2.4. Model building   

Model building was performed with *Phenix* (Liebschner *et al.*, 2019 ▸). The density map from *RELION* was density-modified with *resolve_cryo_em* to improve the interpretability of features (Terwilliger *et al.*, 2020 ▸). *Map_symmetry* was used to generate noncrystallographic symmetry matrices from the map and *map_box* was used to extract the unique part of the map. Chain *A* of PDB entry 3dkt (Sutter *et al.*, 2008 ▸) was fitted into the map with *dock_in_map*. The five neighboring subunits were added to the model to prevent atom clashes between adjacent subunits during refinement. Iterative rounds of *real_space_refine* (Afonine *et al.*, 2018 ▸) and manual model adjustment in *Coot* (Emsley *et al.*, 2010 ▸) led to the final model [Supplementary Fig. S1(*g*)] (Table 1 ▸). Water molecules were added using *douse*. Fivefold and threefold axis channels were analyzed with *Channel Annotation Package* (*CHAP*) to generate plots of channel radius and hydrophobicity (Klesse *et al.*, 2019 ▸).

## 3. Results   

### 3.1. Overall structure and thermostability   

The high resolution of the cryo-EM density map allowed the unambiguous assignment of nearly all of the amino-acid residues of *T. maritima* encapsulin (TmEnc) [Figs. 1 ▸(*b*)–1 ▸(*f*), Table 1 ▸]. The density for aromatic residues contained holes, which is characteristic of high-resolution cryo-EM maps [Figs. 1 ▸(*c*) and 1 ▸(*d*)]. The model built into the cryo-EM density map is consistent with the previous crystal structure, with a root-mean-square deviation of 0.632 Å as calculated using the *match* command in *UCSF Chimera* (Pettersen *et al.*, 2004 ▸). Nevertheless, many side chains are resolved more clearly, illustrating the interactions that stabilize the overall structure. For example, two prolines were identified in the *cis* conformation and were misidentified as *trans*-prolines in the crystal structure [Figs. 1 ▸(*e*) and 1 ▸(*f*)]. Because the side chains are better resolved in this structure, it is possible to see how side-chain interactions may contribute to the extreme thermostability of TmEnc, particularly when compared with the recent structures of encapsulins from other bacterial species such as *Mycolicibacterium hassiacum* (MhEnc; PDB entry 6i9g; Lončar *et al.*, 2020 ▸), *Synechococcus elongatus* (SeEnc; PDB entry 6x8m; Nichols *et al.*, 2020 ▸) and *Quasi­bacillus thermotolerans* (QsEnc; PDB entry 6nj8; Giessen *et al.*, 2019 ▸). Comparing their amino-acid contents, TmEnc has a higher percentage of charged residues, particularly glutamates and lysines, than the other encapsulins (Supplementary Table S1). It also contains more aromatic residues than MhEnc and SeEnc, with the increase mostly attributable to an increased number of phenylalanines (QsEnc has about the same percentage). The increased content of charged and aromatic residues is compensated by a decrease in the content of polar uncharged and aliphatic amino acids. The structure of TmEnc is extensively stabilized by 22 ion pairs; of these, only four out of a total of ten ion pairs are conserved in MhEnc and only two out of a total of 13 ion pairs are conserved in SeEnc. The N- and C-terminal ends are also shorter than other encapsulins, anchored more closely to the structure and more structured, as shown by their resolvability in the density map. These trends are consistent with the trends for thermostable proteins (Vieille & Zeikus, 2001 ▸).

One region with additional stabilizing interactions is the E-loop interface. The E-loop contains a β-strand (residues 66–70) that forms an intermolecular β-sheet with a β-strand from another monomer (residues 75–79) to form a tightly bound dimer [Fig. 1 ▸(*g*)]. The residues that form the intermolecular β-sheet are highly conserved between TmEnc and MhEnc in both sequence and structure. The major difference is in the number of hydrophobic and aromatic contacts: TmEnc contains 100 hydrophobic and 36 aromatic contacts in this interface, whereas MhEnc contains only 36 hydrophobic and zero aromatic contacts (Jubb *et al.*, 2017 ▸). These additional interactions may contribute to the thermostable nature of TmEnc relative to MhEnc. The binding free energy of the residues in the E-loop interface was estimated using the *HawkDock* web server and one of the largest contributors to the binding energy of the E-loop interface of TmEnc is Trp45, which forms a strong intermolecular cation–π interaction with Arg70 [Fig. 1 ▸(*g*)] (Weng *et al.*, 2019 ▸). Trp45 is not conserved in MhEnc and there are no other residues that compensate for the missing cation–π interaction. Arg70 also forms an intermolecular ion pair with Glu77, which further adds to its binding energy. *HawkDock* estimates that Arg70 has a binding free energy of −14.55 kcal mol^−1^ in TmEnc and −6.5 kcal mol^−1^ in MhEnc, which demonstrates the stabilizing effect of Trp45 in TmEnc. Taken together, the thermostability of TmEnc appears to be the result of additional hydrophobic and aromatic interactions in the E-loop interface.

### 3.2. Putative iron-transport channels   

TmEnc natively encapsulates a ferritin-like protein with ferroxidase activity (Sutter *et al.*, 2008 ▸). Previously, three major routes were identified for possible iron transport: channels at the fivefold axis, at the threefold axis and between two adjacent subunits, but these channels were not analyzed in more detail (Sutter *et al.*, 2008 ▸; Williams *et al.*, 2018 ▸). The resolution of the cryo-EM map allows a more detailed inspection of putative iron-transport channels in the TmEnc shell.

**Figure 2:**
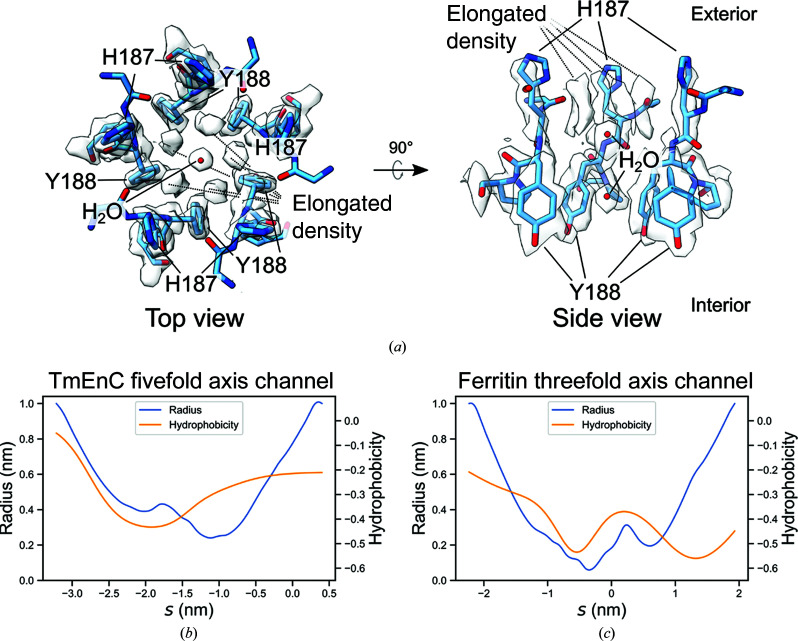

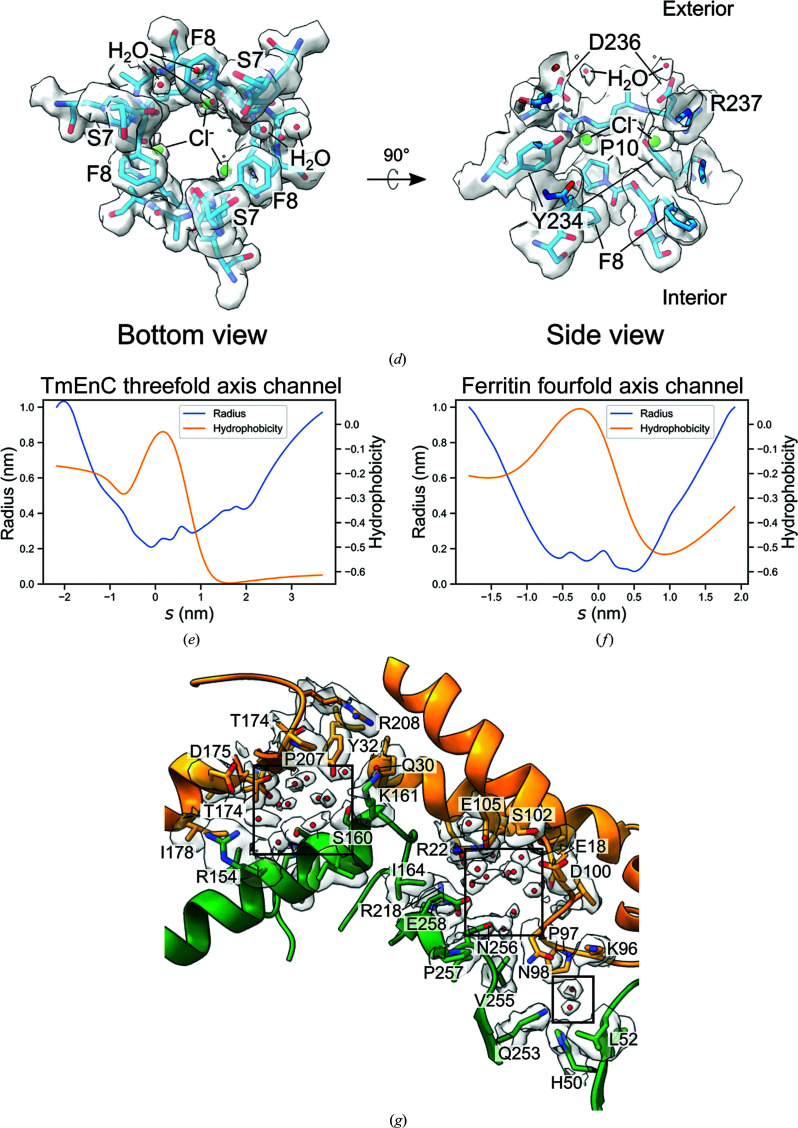
Putative iron-transport channels. (*a*) The fivefold axis channel consists of His187 and Tyr188. The left image shows the top view looking at the channel from the exterior into the interior of TmEnc and the right image is a cut-through side view. Density corresponding to two solvent molecules or ions is present in the channel. Above the two molecules are fivefold-symmetric elongated densities that could not be identified confidently. (*b*, *c*) Radius and hydrophobicity plots using data from *CHAP* of (*b*) the TmEnc fivefold axis channel and (*c*) the ferritin threefold axis channel as a function of probe position *s*. More negative *s* values correspond to positions closer to the exterior and vice versa. Hydrophobicity is measured with the Wimley–White scale (Wimley & White, 1996 ▸). Scales for each graph are identical for comparison. (*d*) The left image shows the threefold axis channel as viewed from the interior of TmEnc looking outwards. The right image is a cut-through side view. The threefold axis channel is formed by residues Asp236, Arg237, Pro10, Tyr234 and Phe8. Density for solvent (red spheres) and chloride ions (green spheres) is present in the channel (Skitchenko *et al.*, 2020 ▸). (*e*, *f*) Radius and hydrophobicity plots of (*e*) the TmEnc threefold axis channel and (*f*) the ferritin fourfold axis channel as in (*b*) and (*c*). (*g*) Channels in the dimer interface are indicated in boxes, with water molecules shown as red spheres. Residues within 5 Å of the channel and their side-chain densities from the cryo-EM map are shown. Red spheres represent water molecules.

The fivefold axis channel is formed by five histidine residues that point towards the exterior (His187) and five tyrosine residues that point towards the interior (Tyr188) [Fig. 2 ▸(*a*)]. The five histidine residues on the exterior of the channel are intriguing because multiple histidines can coordinate metals such as iron and nickel, as employed in immobilized metal-affinity chromatography purification of recombinant histidine-tagged proteins (Block *et al.*, 2009 ▸). The density for His187 is not well resolved in the structure, suggesting that it may be conformationally dynamic. Despite this, His187 is not conserved in other encapsulins, leaving the function of this residue unclear. The radius of the fivefold axis channel is about 2.4 Å at the smallest point, which could accommodate Fe^2+^ (ionic radius of 75 pm) [Fig. 2 ▸(*b*)] (Shannon, 1976 ▸). Compared with the threefold axis channel of ferritin, which is known to allow iron transport, the fivefold axis channel in TmEnc is larger in radius but is more hydrophobic [Figs. 2 ▸(*b*) and 2 ▸(*c*)] (Takahashi & Kuyucak, 2003 ▸; Chandramouli *et al.*, 2016 ▸). Density for two solvent molecules or ions in the fivefold axis channel suggests that iron transport through this channel could be slowed by the hydrophobic sides of the tyrosine side chain, in contrast to the ferritin threefold axis channel, which contains polar residues [Fig. 2 ▸(*a*)] (Pozzi *et al.*, 2015 ▸). Because it lacks polar residues to reduce the energetic barrier of desolvation, the fivefold axis channel may allow iron transport, but the rate is probably slower than free diffusion. Additionally, above the two molecules in the fivefold axis channel is elongated density with fivefold symmetry due to the symmetric cryo-EM 3D reconstruction. The identity of this elongated density could not be assigned confidently.

The threefold axis channel is large enough to accommodate an iron ion, with a radius of 2.9 Å at the smallest point, but the channel has three phenylalanine residues (Phe8) on the interior side that seem to preclude rapid iron transport into the encapsulin [Fig. 2 ▸(*d*)]. The threefold channel of TmEnc has similar hydrophobicity to the fourfold axis channel of ferritin, which is lined with hydrophobic residues and does not transport iron [Figs. 2 ▸(*e*) and 2 ▸(*f*)] (Takahashi & Kuyucak, 2003 ▸). Density for solvent and ion molecules is present in the threefold axis channel of TmEnc, which may be trapped and prevented from entering the interior of the compartment by the hydrophobic Phe8 residues. Therefore, the structure of the threefold axis channel suggests that it either blocks or only allows a slow rate of iron transport. Unlike the fivefold and threefold channels, the channels between a dimer consist only of hydro­philic residues and are not occluded by hydrophobic areas [Fig. 2 ▸(*g*)]. Two of the channels have a radius of 2.0 Å and the last has a radius of 1.2 Å, which are plausible sizes for iron-ion transport when compared with the 0.6 Å radius of the ferritin threefold axis channel. Taken together, we hypothesize that the fivefold axis channel and the dimer channels allow the transport of iron, but the threefold axis channel is unlikely to be permeable to iron.

### 3.3. Flavin ligand   

**Figure 3:**
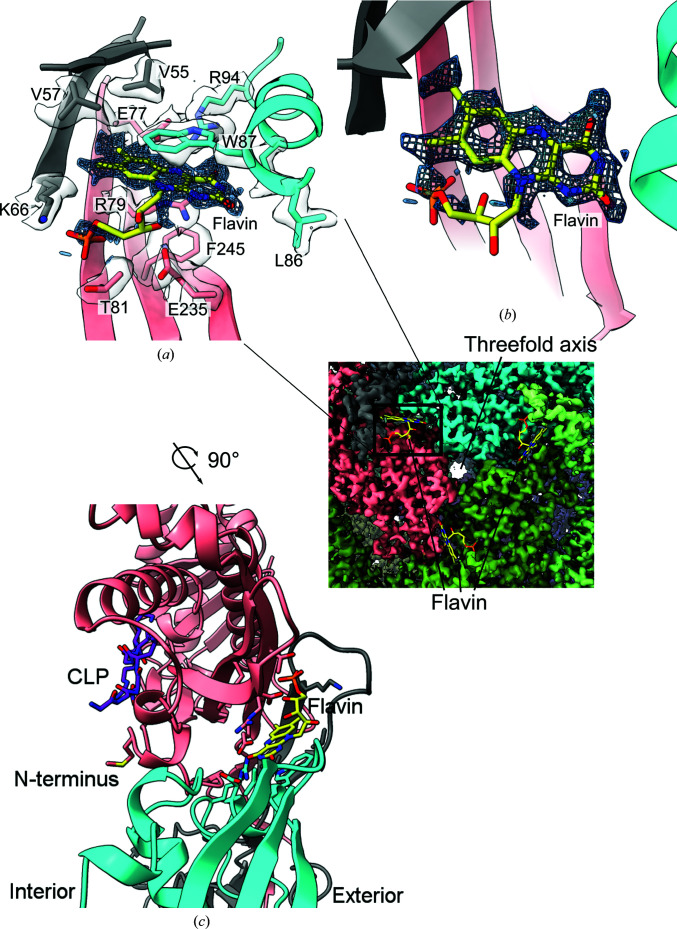
(*a*) Density for a tricyclic ligand was found in the cryo-EM map, consistent with flavin mononucleotide (FMN). Amino acids within 5 Å that form the binding pocket are labeled. Only density for FMN and the amino-acid side chains is shown for clarity. Different colors correspond to different subunits. The inset shows three flavin-binding sites on the exterior of TmEnc around the threefold axis. (*b*) An overhead view of the flavin density and the modeled FMN with the surrounding amino acids hidden. (*c*) The view from (*a*) is rotated 90° clockwise along the *z* axis pointing out of the page and shows that the flavin-binding pocket on the exterior is on the opposite side to the cargo-loading peptide (CLP) and N-terminus in the interior of TmEnc. The cargo protein is not present in the cryo-EM structure, so the CLP from the crystal structure was aligned and placed in the cryo-EM structure. The distance between the centroids of the FMN and CLP is 21.4 Å, which is slightly less than the thickness of the TmEnc compartment.

Inspection of density on the exterior of TmEnc surrounding the threefold axis revealed a tricyclic ring consistent with a flavin ligand [Figs. 3 ▸(*a*) and 3 ▸(*b*)]. This flavin is the first small-molecule ligand to be seen in an encapsulin structure and explains the yellow color of purified TmEnc. The UV–Vis spectrum of TmEnc has peaks at 360 and 450 nm, consistent with the spectra of flavins [Supplementary Fig. S1(*c*)] (Schwinn *et al.*, 2020 ▸). Although the exact identity of the flavin ligand is ambiguous, flavin mononucleotide (FMN) seems to be consistent with the density and has been modeled into the structure. The flavin-binding site consists of residues of three different subunits, but these three subunits are distinct from the trimer that forms the threefold axis [Fig. 3 ▸(*a*), inset]. The structure was entered into the *CSM-lig* web server, which predicted a −log_10_(*K*_d_|*K*_i_) affinity of 13.1, which is higher than those of FMN-binding fluorescent protein and FMN-binding protein from *Desulfovibrio vulgaris* (predicted affinities of 10.1 and 9.6, respectively; Pires & Ascher, 2016 ▸; Möglich & Moffat, 2007 ▸; Suto *et al.*, 2000 ▸). This high affinity is also shown by the retention of the flavin ligand during the purification of TmEnc from the *E. coli* lysate through the 80°C incubation step and into the final cryo-EM sample (see Section 2). Trp87 forms an aromatic stacking interaction with the flavin and is likely to be important for flavin binding [Fig. 3 ▸(*a*)]. Using an *HMMER* search for 150 homologous proteins, the *ConSurf* web server showed that Trp87 was 72% conserved (Ashkenazy *et al.*, 2016 ▸). When the TmEnc sequence was aligned with its broader protein family Linocin_M18 (Pfam PF04454_rp55), Trp is only found at this position in 10% of sequences. Instead, the most common amino acids at this position are Arg, Glu and Ala, which appear in 26%, 17% and 13% of sequences, respectively. Using the *mCSM-lig* web server to predict the change in binding affinity with mutation, W87R, W87E and W87A are all predicted to be destabilizing mutations for flavin binding relative to Trp (Pires *et al.*, 2016 ▸). The lack of conservation of Trp87 in the broader encapsulin alignment suggests that flavin binding is conserved in closely related encapsulins but may not be conserved in more distantly related encapsulins, perhaps those with different types of cargo proteins and functions. For example, the cargo protein of the MhEnc encapsulin is a DyP protein and does not have Trp87, and appears not to have a flavin-binding site (Lončar *et al.*, 2020 ▸). Another interesting aspect of the flavin-binding site is that it is directly opposite the cargo-loading peptide-binding site on the interior of TmEnc, although the significance of this placement is not clear [Fig. 3 ▸(*b*)]. Although the cryo-EM structure of TmEnc revealed this high-affinity flavin-binding site, the role of the flavin in the context of the function of encapsulin needs further investigation.

## 4. Discussion   

The high-resolution structure of TmEnc presented here allows detailed inspection of the structural origins of thermostability, putative iron-transport channels and a newly discovered flavin-binding site. TmEnc contains many of the patterns associated with thermostable proteins versus their mesophilic counterparts, including more charged and hydrophobic residues, many ion pairs and shorter, more structured N- and C-termini. The increased thermostability of TmEnc relative to other encapsulins could be a result of additional hydrophobic and aromatic interactions in the E-loop interface. A cation–π intermolecular interaction seems to be a major contributor to the stability of this interface. This finding has implications for engineering protein–protein interfaces, as it suggests that complementary aromatic–aromatic stacking and cation–π interactions allow more stable interfaces beyond what is possible with just ionic, polar and hydrophobic interactions.

TmEnc natively encapsulates a ferritin-like-protein that stores iron, and a major question is how iron is transported from the exterior to the interior of the compartment. TmEnc has several channels that may allow iron transport: the fivefold axis channel, the threefold axis channel and several channels in the dimer interface. The fivefold axis channel is more likely to transport iron than the threefold axis channel because it is less hydrophobic. Nonetheless, the bottom of the fivefold axis channel consists of the hydrophobic sides of tyrosine side chains, suggesting that iron may not be able to freely diffuse through. This is consistent with a report that the encapsulated ferritin-like protein has an entry site that prevents rapid passage to the ferroxidase center (Piergentili *et al.*, 2020 ▸). Iron transport may be regulated such that iron ions are conducted along a defined pathway in TmEnc towards the ferritin-like protein, as opposed to random diffusion.

This structure provides the first direct evidence for a small-molecule ligand-binding site in an encapsulin nanocompartment. This flavin ligand raises the possibility that the encapsulin shell itself could directly be involved in regulating iron storage and release, instead of just merely providing a compartmental function. For comparison, the addition of free flavins to ferritin is known to promote the release of iron (Satoh *et al.*, 2019 ▸). This observation suggests that one possible role of the flavin ligand is to serve as an electron sink for iron oxidation or as a donor for iron reduction. This behavior may also explain how encapsulins with an Flp cargo help cells to resist oxidative stress, as flavins are known to be free-radical scavengers (McHugh *et al.*, 2014 ▸; Sinha *et al.*, 2020 ▸). Because *T. maritima* can use iron(III) as a terminal electron acceptor in metabolism, TmEnc and its associated Flp may be involved in recycling iron(II) back to iron(III); therefore, TmEnc could be intimately involved in the energy metabolism of *T. maritima* (Vargas *et al.*, 1998 ▸). The identification of the flavin ligand in TmEnc lays the groundwork for a biochemical investigation of these newly hypothesized roles for Flp-containing encapsulin proteins.

This data set was collected in super-resolution mode with a Gatan K3 detector, which enabled the reconstruction to surpass the physical Nyquist limit (2.0 Å resolution, compared with the 2.158 Å Nyquist limit from a 1.079 Å physical pixel size; Feathers *et al.*, 2019 ▸). Because the resolution is beyond the physical Nyquist limit, the resolution of this data set is most probably limited by the pixel size of the micrographs and not by the sample, as is the case for most macromolecules in cryo-EM. If another data set was collected at higher magnification, the resolution of TmEnc could surpass 2 Å (Cheng *et al.*, 2015 ▸). To test the robustness of TmEnc in cryo-EM, random particle subsets ranging from 91 to 185 459 particles were refined in *RELION*. Only 363 particles were needed to reach <3 Å, 5796 for <2.5 Å and 46 365 particles for 2.11 Å resolution, indicating a high degree of homogeneity (Supplementary Fig. S2). Also, the resulting Henderson–Rosenthal plot has a relatively low *B* factor of 51 Å^2^ (Rosenthal & Henderson, 2003 ▸). Due to the ease of purification, *in vitro* stability, favorable cryo-EM behavior and high symmetry, we propose that TmEnc could be used as a model protein to test the resolution limits of cryo-EM structure determination.

## 5. Data availability   

The cryo-EM structure of TmEnc has been deposited in the Protein Data Bank as entry 7kq5. The cryo-EM density maps can be accessed from the Electron Microscopy Data Bank as entry EMD-22992. Raw movie files are available from the Electron Microscopy Public Image Archive as entry EMPIAR-10674.

## Supplementary Material

PDB reference: *T. maritima* encapsulin, 7kq5

EMDB reference: *T. maritima* encapsulin, EMD-22992

Supplementary Figures and Table. DOI: 10.1107/S2052252521001949/pw5017sup1.pdf

## Figures and Tables

**Table 1:** Data-collection, map-reconstruction and model-refinement statistics

Data collection	
Electron microscope	Titan Krios
Electron detector	Gatan K3
Voltage (keV)	300
Defocus range (µm)	0.8–2.5
Original pixel size (Å)	0.5395 (super-resolution)
Electron exposure (e^−^ Å^−2^)	33.5
Images	2772
Map reconstruction	
Final particles	185459
Final pixel size (Å)	0.73
Applied symmetry	*I*3
Resolution (0.143 FSC threshold) (Å)	2.0
*B* factor (Å^2^)	−69
Model refinement	
R.m.s.d., bond lengths (Å)	0.005
R.m.s.d., angles (°)	0.852
*MolProbity* score	1.58
Clashscore	4.89
Ramachandran plot	
Outliers (%)	0.51
Allowed (%)	4.18
Favored (%)	95.31
Ramachandran plot *Z*-score, r.m.s.d.	
Overall (*N* = 1578)	1.51 (0.19)
Helix (*N* = 528)	0.57 (0.19)
Sheet (*N* = 396)	1.03 (0.23)
Loop (*N* = 654)	1.16 (0.23)
Rotamer outliers (%)	0.93
*CaBLAM* outliers (%)	1.92
CC (mask)	0.85
CC (box)	0.48
CC (peaks)	0.38
CC (volume)	0.81
Mean CC for ligands	0.67

## References

[bb1] Afonine, P. V., Poon, B. K., Read, R. J., Sobolev, O. V., Terwilliger, T. C., Urzhumtsev, A. & Adams, P. D. (2018). *Acta Cryst.* D**74**, 531–544. 10.1107/S2059798318006551 PMC6096492 29872004

[bb2] Ashkenazy, H., Abadi, S., Martz, E., Chay, O., Mayrose, I., Pupko, T. & Ben-Tal, N. (2016). *Nucleic Acids Res.* **44**, W344–W350. 10.1093/nar/gkw408 PMC4987940 27166375

[bb3] Block, H., Maertens, B., Spriestersbach, A., Brinker, N., Kubicek, J., Fabis, R., Labahn, J. & Schäfer, F. (2009). *Methods Enzymol.* **463**, 439–473. 10.1016/S0076-6879(09)63027-5 19892187

[bb4] Cassidy-Amstutz, C., Oltrogge, L., Going, C. C., Lee, A., Teng, P., Quintanilla, D., East-Seletsky, A., Williams, E. R. & Savage, D. F. (2016). *Biochemistry*, **55**, 3461–3468. 10.1021/acs.biochem.6b00294 27224728

[bb5] Chandramouli, B., Bernacchioni, C., Di Maio, D., Turano, P. & Brancato, G. (2016). *J. Biol. Chem.* **291**, 25617–25628. 10.1074/jbc.M116.748046 PMC5207259 27756844

[bb6] Cheng, Y., Grigorieff, N., Penczek, P. A. & Walz, T. (2015). *Cell*, **161**, 438–449. 10.1016/j.cell.2015.03.050 PMC4409659 25910204

[bb7] Emsley, P., Lohkamp, B., Scott, W. G. & Cowtan, K. (2010). *Acta Cryst.* D**66**, 486–501. 10.1107/S0907444910007493 PMC2852313 20383002

[bb8] Feathers, J. R., Spoth, K. A. & Fromme, J. C. (2019). *bioRxiv*, 675397.

[bb9] Giessen, T. W., Orlando, B. J., Verdegaal, A. A., Chambers, M. G., Gardener, J., Bell, D. C., Birrane, G., Liao, M. & Silver, P. A. (2019). *eLife*, **8**, e46070. 10.7554/eLife.46070 PMC6668986 31282860

[bb10] Giessen, T. W. & Silver, P. A. (2017). *Nat. Microbiol.* **2**, 17029. 10.1038/nmicrobiol.2017.29 28263314

[bb12] Jones, J. A. & Giessen, T. W. (2021). *Biotechnol. Bioeng.* **118**, 491–505. 10.1002/bit.27564 PMC8182298 32918485

[bb13] Jubb, H. C., Higueruelo, A. P., Ochoa-Montaño, B., Pitt, W. R., Ascher, D. B. & Blundell, T. L. (2017). *J. Mol. Biol.* **429**, 365–371. 10.1016/j.jmb.2016.12.004 PMC5282402 27964945

[bb14] Klesse, G., Rao, S., Sansom, M. S. P. & Tucker, S. J. (2019). *J. Mol. Biol.* **431**, 3353–3365. 10.1016/j.jmb.2019.06.003 PMC6699600 31220459

[bb15] Liebschner, D., Afonine, P. V., Baker, M. L., Bunkóczi, G., Chen, V. B., Croll, T. I., Hintze, B., Hung, L.-W., Jain, S., McCoy, A. J., Moriarty, N. W., Oeffner, R. D., Poon, B. K., Prisant, M. G., Read, R. J., Richardson, J. S., Richardson, D. C., Sammito, M. D., Sobolev, O. V., Stockwell, D. H., Terwilliger, T. C., Urzhumtsev, A. G., Videau, L. L., Williams, C. J. & Adams, P. D. (2019). *Acta Cryst.* D**75**, 861–877. 10.1107/S2059798319011471 PMC6778852 31588918

[bb16] Lončar, N., Rozeboom, H. J., Franken, L. E., Stuart, M. C. A. & Fraaije, M. W. (2020). *Biochem. Biophys. Res. Commun.* **529**, 548–553. 10.1016/j.bbrc.2020.06.059 32736672

[bb17] McHugh, C. A., Fontana, J., Nemecek, D., Cheng, N., Aksyuk, A. A., Heymann, J. B., Winkler, D. C., Lam, A. S., Wall, J. S., Steven, A. C. & Hoiczyk, E. (2014). *EMBO J.* **33**, 1896–1911. 10.15252/embj.201488566 PMC4195785 25024436

[bb18] Möglich, A. & Moffat, K. (2007). *J. Mol. Biol.* **373**, 112–126. 10.1016/j.jmb.2007.07.039 PMC2175523 17764689

[bb19] Morin, A., Eisenbraun, B., Key, J., Sanschagrin, P. C., Timony, M. A., Ottaviano, M. & Sliz, P. (2013). *eLife*, **2**, e01456. 10.7554/eLife.01456 PMC3771563 24040512

[bb20] Nichols, R., LaFrance, B., Phillips, N., Oltrogge, L., Valentin-Alvarado, L., Bischoff, A., Nogales, E. & Savage, D. (2020). *bioRxiv*, 2020.05.24.113720.

[bb21] Nichols, R. J., Cassidy-Amstutz, C., Chaijarasphong, T. & Savage, D. F. (2017). *Crit. Rev. Biochem. Mol. Biol.* **52**, 583–594. 10.1080/10409238.2017.1337709 28635326

[bb22] Pettersen, E. F., Goddard, T. D., Huang, C. C., Couch, G. S., Greenblatt, D. M., Meng, E. C. & Ferrin, T. E. (2004). *J. Comput. Chem.* **25**, 1605–1612. 10.1002/jcc.20084 15264254

[bb23] Piergentili, C., Ross, J., He, D., Gallagher, K. J., Stanley, W. A., Adam, L., Mackay, C. L., Baslé, A., Waldron, K. J., Clarke, D. J. & Marles-Wright, J. (2020). *J. Biol. Chem.* **295**, 15511–15526. 10.1074/jbc.RA120.014502 PMC7667983 32878987

[bb24] Pires, D. E. V. & Ascher, D. B. (2016). *Nucleic Acids Res.* **44**, W557–W561. 10.1093/nar/gkw390 PMC4987933 27151202

[bb25] Pires, D. E. V., Blundell, T. L. & Ascher, D. B. (2016). *Sci. Rep.* **6**, 29575. 10.1038/srep29575 PMC4935856 27384129

[bb26] Pozzi, C., Di Pisa, F., Bernacchioni, C., Ciambellotti, S., Turano, P. & Mangani, S. (2015). *Acta Cryst.* D**71**, 1909–1920. 10.1107/S1399004715013073 26327381

[bb27] Rohou, A. & Grigorieff, N. (2015). *J. Struct. Biol.* **192**, 216–221. 10.1016/j.jsb.2015.08.008 PMC6760662 26278980

[bb28] Rosenthal, P. B. & Henderson, R. (2003). *J. Mol. Biol.* **333**, 721–745. 10.1016/j.jmb.2003.07.013 14568533

[bb29] Satoh, J., Kimata, S., Nakamoto, S., Ishii, T., Tanaka, E., Yumoto, S., Takeda, K., Yoshimura, E., Kanesaki, Y., Ishige, T., Tanaka, K., Abe, A., Kawasaki, S. & Niimura, Y. (2019). *J. Gen. Appl. Microbiol.* **65**, 308–315. 10.2323/jgam.2019.03.001 31281172

[bb30] Scheres, S. H. W. (2012). *J. Struct. Biol.* **180**, 519–530. 10.1016/j.jsb.2012.09.006 PMC3690530 23000701

[bb31] Schwinn, K., Ferré, N. & Huix-Rotllant, M. (2020). *Phys. Chem. Chem. Phys.* **22**, 12447–12455. 10.1039/d0cp01714k 32458897

[bb32] Shannon, R. D. (1976). *Acta Cryst.* A**32**, 751–767.

[bb33] Sinha, T., Naash, M. I. & Al-Ubaidi, M. R. (2020). *Front. Cell. Dev. Biol.* **8**, 861. 10.3389/fcell.2020.00861 PMC7481326 32984341

[bb34] Skitchenko, R. K., Usoltsev, D., Uspenskaya, M., Kajava, A. V. & Guskov, A. (2020). *Bioinformatics*, **36**, 3064–3071. 10.1093/bioinformatics/btaa079 PMC7214031 32022861

[bb35] Suto, K., Kawagoe, K., Shibata, N., Morimoto, Y., Higuchi, Y., Kitamura, M., Nakaya, T. & Yasuoka, N. (2000). *Acta Cryst.* D**56**, 368–371. 10.1107/s0907444900000111 10713530

[bb36] Sutter, M., Boehringer, D., Gutmann, S., Günther, S., Prangishvili, D., Loessner, M. J., Stetter, K. O., Weber-Ban, E. & Ban, N. (2008). *Nat. Struct. Mol. Biol.* **15**, 939–947. 10.1038/nsmb.1473 19172747

[bb37] Takahashi, T. & Kuyucak, S. (2003). *Biophys. J.* **84**, 2256–2263. 10.1016/S0006-3495(03)75031-0 PMC1302792 12668434

[bb38] Terwilliger, T. C., Ludtke, S. J., Read, R. J., Adams, P. D. & Afonine, P. V. (2020). *Nat. Methods*, **17**, 923–927. 10.1038/s41592-020-0914-9 PMC7484085 32807957

[bb39] Tracey, J. C., Coronado, M., Giessen, T. W., Lau, M. C. Y., Silver, P. A. & Ward, B. B. (2019). *Sci. Rep.* **9**, 20122. 10.1038/s41598-019-56533-5 PMC6934571 31882935

[bb40] Vargas, M., Kashefi, K., Blunt-Harris, E. L. & Lovley, D. R. (1998). *Nature*, **395**, 65–67. 10.1038/25720 9738498

[bb41] Vieille, C. & Zeikus, G. J. (2001). *Microbiol. Mol. Biol. Rev.* **65**, 1–43. 10.1128/MMBR.65.1.1-43.2001 PMC99017 11238984

[bb42] Wagner, T., Merino, F., Stabrin, M., Moriya, T., Antoni, C., Apelbaum, A., Hagel, P., Sitsel, O., Raisch, T., Prumbaum, D., Quentin, D., Roderer, D., Tacke, S., Siebolds, B., Schubert, E., Shaikh, T. R., Lill, P., Gatsogiannis, C. & Raunser, S. (2019). *Commun. Biol.* **2**, 218. 10.1038/s42003-019-0437-z PMC6584505 31240256

[bb43] Weng, G., Wang, E., Wang, Z., Liu, H., Zhu, F., Li, D. & Hou, T. (2019). *Nucleic Acids Res.* **47**, W322–W330. 10.1093/nar/gkz397 PMC6602443 31106357

[bb44] Williams, E. M., Jung, S. M., Coffman, J. L. & Lutz, S. (2018). *ACS Synth. Biol.* **7**, 2514–2517. 10.1021/acssynbio.8b00295 30376298

[bb45] Wimley, W. C. & White, S. H. (1996). *Nat. Struct. Mol. Biol.* **3**, 842–848. 10.1038/nsb1096-842 8836100

[bb46] Zivanov, J., Nakane, T. & Scheres, S. H. W. (2020). *IUCrJ*, **7**, 253–267. 10.1107/S2052252520000081 PMC7055373 32148853

