# Supplementary material for: Cryo-EM structure of a thermostable bacterial nanocompartment
Source: IUCrJ. 2021 Apr 2;8(Pt 3):342–50. doi: 10.1107/S2052252521001949 (PMC8086157; doi:10.1107/S2052252521001949)
Supplement: Supplementary file 1 [file m-08-00342-sup1.pdf]

IUCrJ

Volume 8 (2021)

Supporting information for article:

# Cryo-EM structure of a thermostable bacterial nanocompartment

Timothy Wiryaman and Navtej Toor

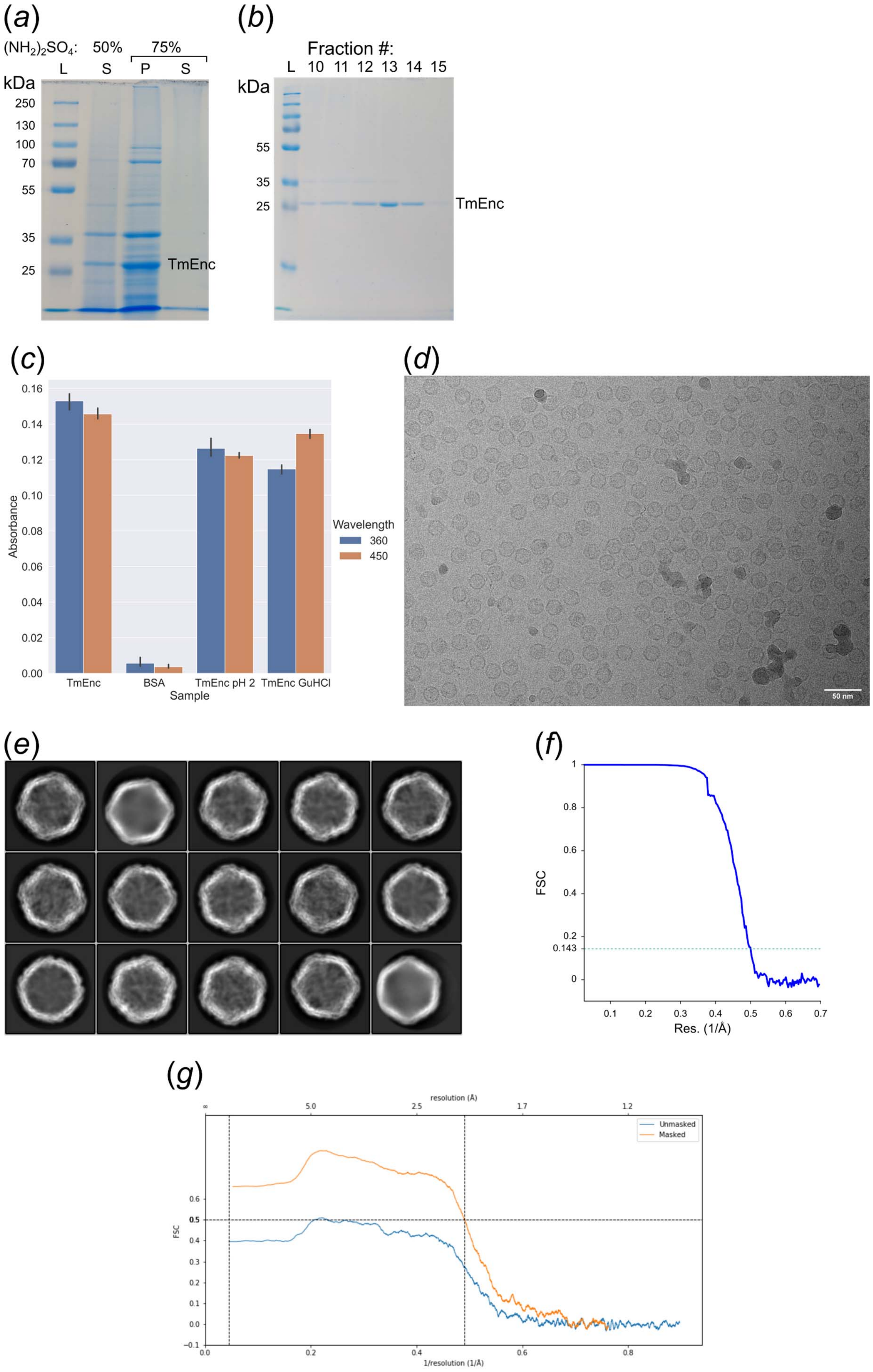
(a)
(NH2)2SO4: 50% 75%
L S P S
kDa
250
130
100
70
55
35
25
TmEnc
(b)
Fraction #:
L 10 11 12 13 14 15
kDa
55
35
25
TmEnc
(c)
Absorbance
0.16
0.14
0.12
0.10
0.08
0.06
0.04
0.02
0.00
TmEnc
BSA
TmEnc pH 2
TmEnc GuHCl
Sample
Wavelength
360
450
(d)
50 nm
(e)
(f)
FSC
1
0.8
0.6
0.4
0.2
0.143
0
0.1 0.2 0.3 0.4 0.5 0.6 0.7
Res. (1/Å)
(g)
resolution (Å)
∞ 5.0 2.5 1.7 1.2
Unmasked
Masked
FSC
0.6
0.5
0.4
0.3
0.2
0.1
0.0
−0.1
0.0 0.2 0.4 0.6 0.8
1/resolution (1/Å)

**Figure S1** (*a*) 10% SDS-polyacrylamide gel of TmEnc during ammonium sulfate precipitation step in purification. Percentages above lanes indicate the ammonium sulfate saturation. S refers to the supernatant and P refers to the pellet after incubating the protein solution with ammonium sulfate and centrifuging. (*b*) 12% SDS-polyacrylamide gel of fractions collected after purifying TmEnc on a HiPrep Sephacryl S-500 column. (*c*) UV-vis absorption at 360 nm and 450 nm for TmEnc, TmEnc diluted in pH 2.0 buffer, TmEnc diluted in 8 M guanidine hydrochloride, and bovine serum albumin (BSA). Error bars are 95% confidence intervals. (*d*) Representative micrograph of TmEnc from cryo-EM data collection. The micrograph was low-pass filtered to 20 Å. (*e*) 2D class averages of *crYOLO*-picked particles calculated by *RELION*. (*f*) Fourier shell correlation of two half-maps refined independently in *RELION*. (*g*) Map-to-model Fourier shell correlation after refinement in *PHENIX*.

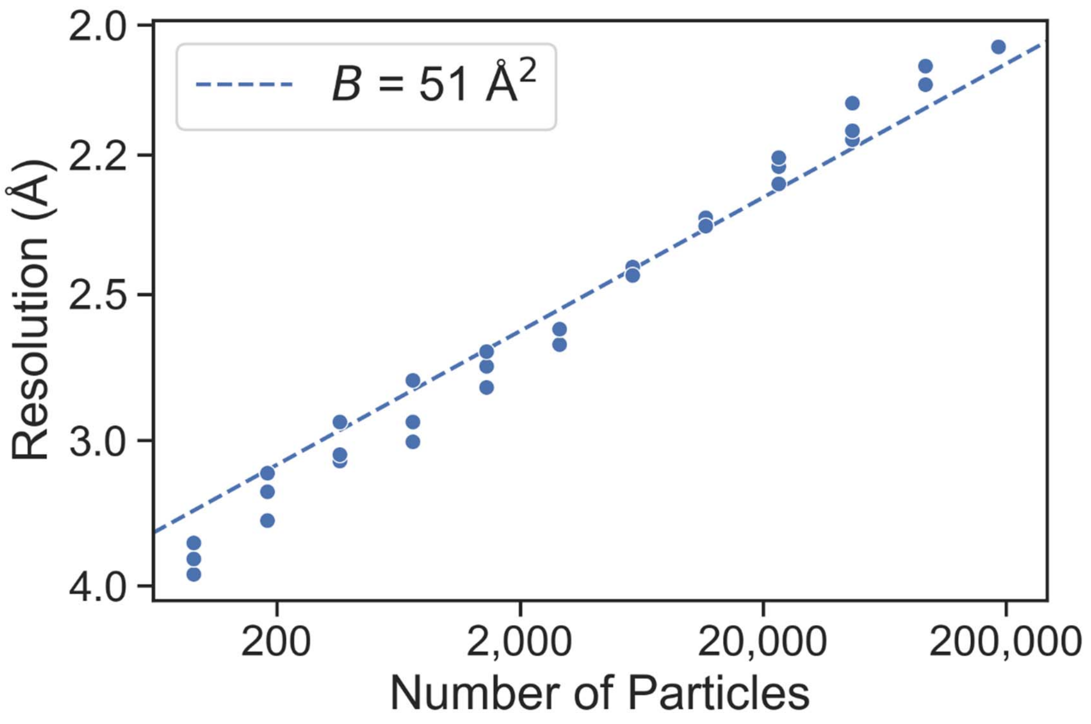


**Figure S2** Particle subsets were taken from the final polished particles and refined in RELION. The resolution as a function of the number of particles is shown on a Henderson-Rosenthal *B*-factor plot. The estimated *B*-factor from linear regression is 51.3 Å$^2$.

**Table S1** Amino acid content, expressed in percentages, of TmEnc, MhEnc, SeEnc, and QsEnc.

| Name | TmEnc | MhEnc | SeEnc | QsEnc |
|---|---|---|---|---|
| Uniprot Accession | Q9WZP2 | K5BEG2 | Q55032 | A0A0F5HPP7 |
| PDB ID | 7KQ5 | 3I9G | 6X8M | 6NJ8 |
| A | 4.53 | 10.19 | 7.52 | 6.03 |
| C | 1.13 | 0.75 | 0.98 | 0.35 |
| D | 5.66 | 8.68 | 5.56 | 6.74 |
| E | 13.21 | 6.79 | 5.88 | 8.16 |
| F | 5.66 | 2.64 | 2.29 | 5.32 |
| G | 7.17 | 6.42 | 5.23 | 6.03 |
| H | 0.75 | 2.64 | 1.96 | 2.84 |
| I | 6.42 | 5.66 | 4.90 | 6.03 |
| K | 7.17 | 2.26 | 2.61 | 3.90 |
| L | 12.08 | 9.43 | 12.09 | 10.28 |
| M | 0.38 | 0.38 | 1.31 | 3.19 |
| N | 2.64 | 1.51 | 4.90 | 5.67 |
| P | 3.77 | 5.66 | 6.21 | 4.26 |
| Q | 1.89 | 2.26 | 4.58 | 3.90 |
| R | 6.79 | 7.17 | 7.84 | 6.03 |
| S | 4.53 | 6.79 | 5.56 | 5.67 |
| T | 4.91 | 7.17 | 6.54 | 3.90 |
| V | 7.17 | 9.43 | 9.15 | 7.45 |
| W | 1.89 | 1.13 | 1.31 | 0.71 |
| Y | 2.26 | 3.02 | 3.59 | 3.55 |
| Negative (D+E) | 18.87 | 15.47 | 11.44 | 14.89 |
| Positive (H+K+R) | 14.72 | 12.08 | 12.42 | 12.77 |
| Charged total (D+E+H+K+R) | 33.58 | 27.55 | 23.86 | 27.66 |
| Polar uncharged (S+T+N+Q) | 13.96 | 17.74 | 21.57 | 19.15 |
| Aliphatic/hydrophobic (A+I+L+M+V) | 30.57 | 35.09 | 34.97 | 32.98 |
| Aromatic (F+W+Y) | 9.81 | 6.79 | 7.19 | 9.57 |
